# The Relationship Between Specific Pavlovian Instrumental Transfer and Instrumental Reward Probability

**DOI:** 10.3389/fpsyg.2015.01697

**Published:** 2015-11-17

**Authors:** Emilio Cartoni, Tania Moretta, Stefano Puglisi-Allegra, Simona Cabib, Gianluca Baldassarre

**Affiliations:** ^1^Laboratory of Computational Embodied Neuroscience, Institute of Cognitive Sciences and Technologies, National Research Council of ItalyRome, Italy; ^2^Dipartimento di Psicologia, Sapienza - Università di RomaRome, Italy; ^3^Dipartimento di Psicologia Generale, Università di PadovaPadua, Italy; ^4^Dipartimento di Psicologia and Centro Daniel Bovet, Sapienza - Università di RomaRome, Italy; ^5^Fondazione Santa Lucia, Istituto di Ricovero e Cura a Carattere ScientificoRome, Italy

**Keywords:** Pavlovian instrumental transfer, instrumental contingency, reward probability, human PIT, specific PIT

## Abstract

Goal-directed behavior is influenced by environmental cues: in particular, cues associated with a reward can bias action choice toward actions directed to that same reward. This effect is studied experimentally as specific Pavlovian-instrumental transfer (specific PIT). We have investigated the hypothesis that cues associated to an outcome elicit specific PIT by rising the estimates of reward probability of actions associated to that same outcome. In other words, cues reduce the uncertainty on the efficacy of instrumental actions. We used a human PIT experimental paradigm to test the effects of two different instrumental contingencies: one group of participants had a 33% chance of being rewarded for each button press, while another had a 100% chance. The group trained with 33% reward probability showed a stronger PIT effect than the 100% group, in line with the hypothesis that Pavlovian cues linked to an outcome work by reducing the uncertainty of receiving it. The 100% group also showed a significant specific PIT effect, highlighting additional factors that could contribute to specific PIT beyond the instrumental training contingency. We hypothesize that the uncertainty about reward delivery due to testing in extinction might be one of these factors. These results add knowledge on how goal-directed behavior is influenced by the presence of environmental cues associated with a reward: such influence depends on the probability that we have to reach a reward, namely when there is less chance of getting a reward we are more influenced by cues associated with it, and vice versa.

## 1. Introduction

It has long been known that cues associated with a rewarding outcome can elicit and intensify actions directed to obtain that same outcome. This effect can be studied experimentally in a paradigm called specific Pavlovian instrumental transfer (specific PIT). In a typical specific PIT experiment, a participant is first trained to associate two cues with two different outcomes: for example, to associate two different images (Pavlovian conditioned stimulus, CS) each with the delivery of a different reward (e.g., chocolate and popcorns). Then, the participant is trained to make two actions to get these two rewards: for example, to press a left button to get chocolate and to press a right button to get popcorns (instrumental training). In a final test phase, the participant can again press these buttons in extinction (no reward is delivered) but sometimes one of the two images (CS) is displayed. What will happen is that during the image display the participant will press the button corresponding to the same outcome of the image more than the other button. In other words, in specific PIT a Pavlovian cue associated with food (or another reward) selectively increases instrumental actions directed to the same food. This occurs despite the fact that no explicit training of the instrumental actions in the presence of Pavlovian cues is performed.

This PIT effect can play a critical role in regulating goal-directed behavior in different situations of life, ranging from advertising to drug addiction. For example, the vision of a McDonald sign might encourage a person to buy a portion of potato chips. In this case, the McDonald sign might be thought as a CS associated with potato chips that promotes the instrumental action of buying that food. PIT is also relevant for drug addiction as drug-related cues can be a threat to self-control and often lead to relapse after treatment (Hogarth et al., 2007; Belin et al., 2009).

While there is increasing progress on the study of specific PIT neural substrates and mechanisms (Laurent et al., 2014), it is not yet clear how specific PIT works at the functional level and what its adaptive function and evolutionary significance is. Why do Pavlovian cues influence our instrumental behavior through the specific PIT effect? Which are the factors that mediate this effect? It has been proposed that Pavlovian cues elicit specific PIT by signalling an increased efficacy of the instrumental action (Cartoni et al., 2013; Hogarth et al., 2013, 2014; Hogarth and Troisi, 2015).

In Cartoni et al. (2013), we advanced an hypothesis and a computational model on how PIT might bias instrumental behavior by affecting different components of action evaluation. In particular, we modeled one of the possibilities of how specific PIT might be linked to the efficacy of the instrumental action. We suggested that the specific PIT effect is elicited by the presence of an outcome-associated cue that increases the estimate of the probability of reaching that outcome. In other words, if an instrumental response was usually rewarded 33% of the time, the presence of the cue makes the participant think that the chances of being rewarded are now higher (e.g., 50%). This led us to the hypothesis that instrumental responses that are continuously rewarded (100% chance of receiving the reward) cannot be augmented further by the presence of a cue associated with the same reward.

In the study reported here, we tested this hypothesis on specific PIT by contrasting specific PIT effects in two groups of subjects: during instrumental training, one was rewarded with 100% reward chance for each instrumental action (button presses), while another was rewarded only with 33% chance. Other PIT studies have manipulated the instrumental schedules in the past (Meltzer and Hamm, 1974a, 1978; Edgar et al., 1981; Lovibond, 1983; Holland, 2004; Wiltgen et al., 2012, see Section 4); however, to date we are not aware of any work either with humans or animals that has directly manipulated the instrumental contingency during training to see how the size of specific PIT varies as a function of the instrumental probabilities of obtaining an outcome. The closest manipulations we could find were those by Trick et al. (2011), which used Pavlovian stimuli trained with different contingencies and Hogarth et al. (2014) where the participants expectations about instrumental contingency during PIT test were manipulated by verbal instructions. However, none of these directly manipulated the reward probabilities of the instrumental training.

According to our hypothesis, we expected to find a stronger specific PIT effect in the low-probability group (33%) than in the high-probability one (100%); in this latter group the outcome probability was already at maximum, so we expected a minimal or absent specific PIT effect as the estimate of the probability of being rewarded could not be further augmented by the cues. Experimental results confirmed that specific PIT was stronger for participants trained with a lower probability to obtain the outcome by instrumental action. However, a significant specific PIT effect was also found in the high-probability group, despite the fact that the trained reward probability was at maximum. We hypothesize that the uncertainty about reward delivery due to testing in extinction might account for this latter effect.

## 2. Materials and methods

### 2.1. Participants

A sample of 57 volunteer students (32 males) between the ages of 19 and 30 years (mean age = 24.0, *SD* = 2.8) were recruited from the *Sapienza University of Rome*. Following Prévost et al. (2012), the eating attitudes test EAT-26 (Garner et al., 1982) was administered before the experiment to ensure that participants did not have eating disorders. Participants' last meal was on average 2.6 h (*SD* = 2.7) before the experiment start. Written informed consent was obtained from all participants, and the Psychology Ethics Committee of the *Sapienza University of Rome* approved the study. In Prévost et al. (2012), using a paradigm close to the one used here, PIT effects were detected with a sample size of 26 participants. Other studies also used around 20 participants (Bray et al., 2008; Allman et al., 2010; Trick et al., 2011) to detect PIT effects with humans, so we chose as a stopping rule to collect at least 20 participants for each of the two conditions (33% and 100% probability), after considering exclusions due to EAT-26 results or the Assessment Phase. From the initial sample of 57 participants, five were excluded as their results on the EAT-26 suggested a possible eating disorder. Eight participants were also excluded as they failed to answer correctly the questions on the Pavlovian and instrumental reward associations during the Assessment phase. It has already been reported that participants unaware of the contingencies might not express specific PIT, so it is customary to exclude them from the analysis (see Trick et al., 2011; Hogarth et al., 2014; Eder and Dignath, 2015). We then further excluded four participants as outliers after analyzing instrumental training data because they focused almost exclusively on one lever during the instrumental phase. These exclusions left 40 participants for the PIT test analysis, 23 participants for the 33% condition plus 17 participants for the 100% condition.

### 2.2. Stimuli and materials

Visual stimuli were presented by a display (27 × 40 cm) connected with a computer; stimulus presentation and behavioral data acquisition were implemented in Matlab (The Mathworks) with the Psychophysics toolbox (Brainard, 1997; Pelli, 1997; Kleiner et al., 2007). The food rewards were chosen from 14 different sweet and salty snack foods (Bounty, Cipster, Fonzies, Freeky Fries, Kinder bueno, Kinder cereali, Kinder cioccolato, Kit kat, Mars, Milka, Ritz Crispy, Smarties, Tuc, Twix). Participants were asked to provide subjective pleasantness ratings for each snack food clicking with a mouse on an analog scale displayed together with a picture of each snack. The two most pleasant foods for each participant were used as rewards. Two fractal images were employed as conditional stimuli (CS) during the Pavlovian and PIT test phases. Instrumental responses consisted of button presses on a custom response box. The response box had three buttons arranged in an horizontal row each equipped with a spring to make pressing effortful. Only the left and right buttons of the response box were used. Two black squares were presented on the display: these squares became gray to signal when response buttons were available for pressing during each trial. When participants pressed a button, the squares briefly flashed white. Food pictures (rewards) and fractals were displayed between the squares.

### 2.3. Procedure

**Figure 1:**
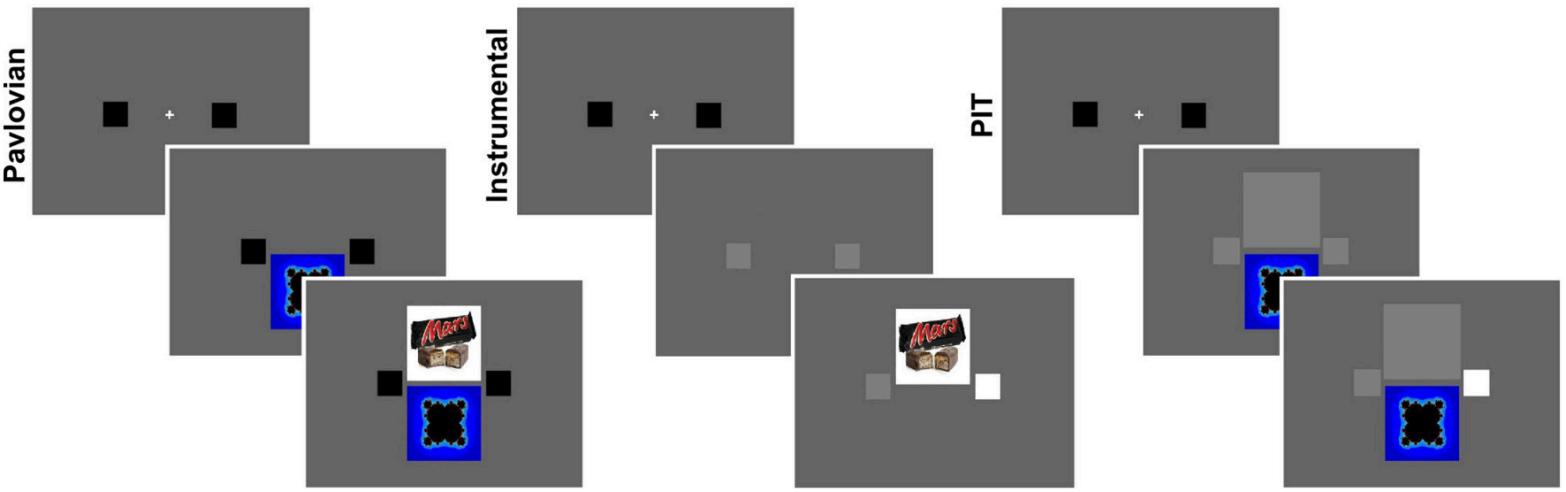
**PIT paradigm**. The three main phases of the experiment: a Pavlovian training phase, an Instrumental training phase, and a PIT test phase. During the Pavlovian phase, a visual cue (conditioned stimulus, CS) was presented at the bottom of the screen for 6 s, which predicted the appearance of a food picture on top for 1 s. Two visual cues were associated with two different food pictures. In the instrumental phase, two small squares, spatially corresponding to two buttons on a response box, both changed color from black to gray for 6 s during which participants could press any button for any number of times to win food rewards. Each button was associated with a different food reward. The two food rewards were the same as the previous phase. Whenever the food reward was won, the corresponding picture was displayed in the upper part of the screen. In the PIT phase the buttons were available but a big gray square covered the space where food reward previously appeared. There were two types of PIT test trials: baseline trials without any visual cue; CUED trials where a visual cue was displayed, associated to the same food as either the left or right button. These three main phases were preceded by a “Taste test” phase and followed by an Assessment phase.

The experiment consisted of three main phases: an instrumental, a Pavlovian, and a PIT phase (see Figure 1). These phases were preceded by a “Taste test” phase and followed by an Assessment phase.

#### 2.3.1. Taste test phase

During this phase, the two squares are black, signaling that no button is available. There are two trials, one for each food chosen for the experiment. On each trial a food image appears and the experimenter then gives a piece of the corresponding food in the hand of the participant for immediate consumption. This phase gives the participants a chance to experience the two foods in a hungry state. This should enhance their motivation for pressing in the subsequent phases and ensure that they “know the value of the reward” in the hungry state.

#### 2.3.2. Pavlovian learning phase

During Pavlovian training, the two squares are always black, signaling that no button is available. For each trial, a fractal image is presented for 6 s. During the last second of this presentation (between 5 and 6 s) a food picture is displayed. Two fractals are used and each fractal paired with one of the foods. The associations between fractal images and foods were randomized across participants. This phase is formed by 20 trials, 10 trials for each fractal. A random duration inter-trial interval is presented for 2–6 s. Before the phase starts participants are told to pay attention to the abstract images that will be presented and that each abstract image will be associated to a different food.

#### 2.3.3. Instrumental learning phase

The instrumental phase is formed by 30 trials, each lasting 6 s with a variable 2–6 s inter trial interval. On each trial the two squares are gray (instead of black), signaling that the corresponding buttons can now be pressed to obtain a reward. Participants are free to choose which button to press (any, none, or both) and how many times to press them on each trial. Button presses are both reinforced either with 33.3% or 100% probability (depending on the participant group). When pressing is reinforced, the corresponding food picture is immediately displayed for 1 s. Food pictures signal that a small piece of that food is won. Pressing a button also makes the corresponding square flash white for 50 ms. Before the phase starts participants are told that food earned during this phase will be given at the end of the experiment. At the end of the phase, a final screen tells the participants how many food pieces they won that far (number of food images displayed) and the corresponding quantity of real food. The quantity of real food won was automatically adjusted so that all participants won an amount of food approximately worth 2 euros.

#### 2.3.4. PIT phase

The PIT phase is similar to the instrumental phase, with two differences. The first difference is that the reward is never displayed: during each trial a big gray square is constantly shown where rewards used to appear, so this phase is carried out in extinction. The extinction is, however, a nominal extinction because as in Hogarth et al. (2007) before the phase starts the participants are told to assume that rewards are still given as before (i.e., they are hidden but still delivered). The second difference is that in some trials a fractal corresponding to the same reward of one of the buttons is displayed. This phase is formed by 46 trials: 15 trials without fractals (baseline trials) randomly mixed with 30 trials with fractals (15 trials for each fractal, referred to as ‘CUED’), plus 1 trial without fractals at the beginning. Before the phase starts, participants are warned that the big gray square will appear so that they may not see when they are rewarded. Participants are also told that the abstract images might appear again, without saying if they are relevant for the current phase or not.

#### 2.3.5. Assessment phase

After completing the PIT phase participants are asked to answer some questions to determine whether they are knowledgeable about the relationships presented in the experiment. Participants answer four two-choice questions, one for each of the fractal-reward and button-reward relationships presented in the experiment. In each question, participants have to choose to which of the two food rewards the fractal or the action was paired. Only those participants who reported the correct pairings were included in the data analysis. The experiment was concluded by a debriefing where participants could ask questions regarding the experiment and the rewards won were given.

## 3. Results

### 3.1. Training

**Figure 2:**
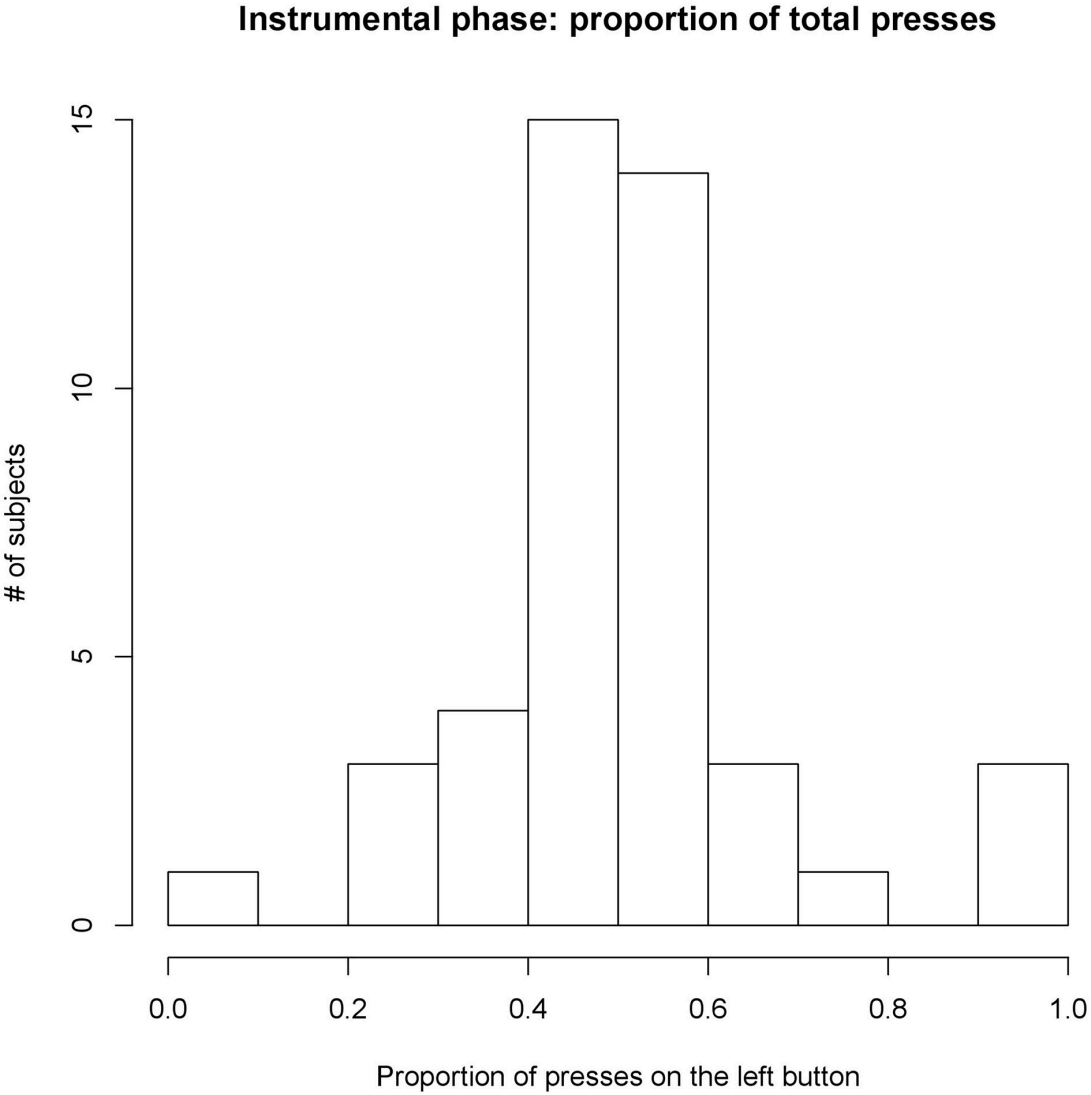
**Proportion of presses allocated to the left button during the instrumental phase**. Most participants allocated roughly 50% of their presses to the left button, thus eperiencing the left and right button and their rewards in a balanced manner. A few participants (four) focused almost exclusively on one button and were considered outliers. Average proportion 52% (*SD* = 17%).

During the instrumental phase, most participants pressed both buttons in a roughly balanced manner, with an average proportion of presses on the left button of 52% (*SD* = 17%, see Figure 2). Despite using subjectively pleasant rewards for both instrumental actions, usually rated at very similar levels on the analog scale, four participants concentrated almost all their efforts on one button only, with only a few presses on the other button. These participants were considered as outliers in their baseline responding and hence excluded from further analysis. One of them explicitly declared during the debrief that he had changed his mind about how much he wanted one of the rewards.

The participants could freely choose how many times to press the buttons, thus the total amount of presses varied among participants, ranging from 54 to 929 total presses over 30 trials. As the rewards were delivered occasionally in the 33% group, participants experienced different degrees of reward probability, depending on the amount of presses and the luck of the draw. Experienced probabilities thus ranged from 20% to 51% with mean 33% (*SD* = 6%). Overall, participants in the 33% group experienced an average of 81 rewards vs. 279 of the 100% group.

### 3.2. PIT test

During the PIT test, the two groups responded with similar baseline rates: 11.2 ± 6.9 vs. 11.6 ± 10.3 presses/trial for the 33% and 100% group, respectively [*t*_(38)_ = 0.19, *p* = 0.85]. To calculate the strength of the PIT effect during the CUED trials, we calculated a PIT strength index for each trial with the following formulas:

(1) $$\begin{array}{l} Proportion=\left(LeftPresses\right)∕\left(LeftPresses+RightPresses\right) \end{array}$$

(2) $$\begin{array}{l} Baseline=\overset{15}{\underset{baselinetrial=1}{\sum }}{Proportion}_{baselinetrial}∕15 \end{array}$$

(3) $$\begin{array}{l} PIT_{strength}=Proportion-Baseline \end{array}$$

**Figure 3:**
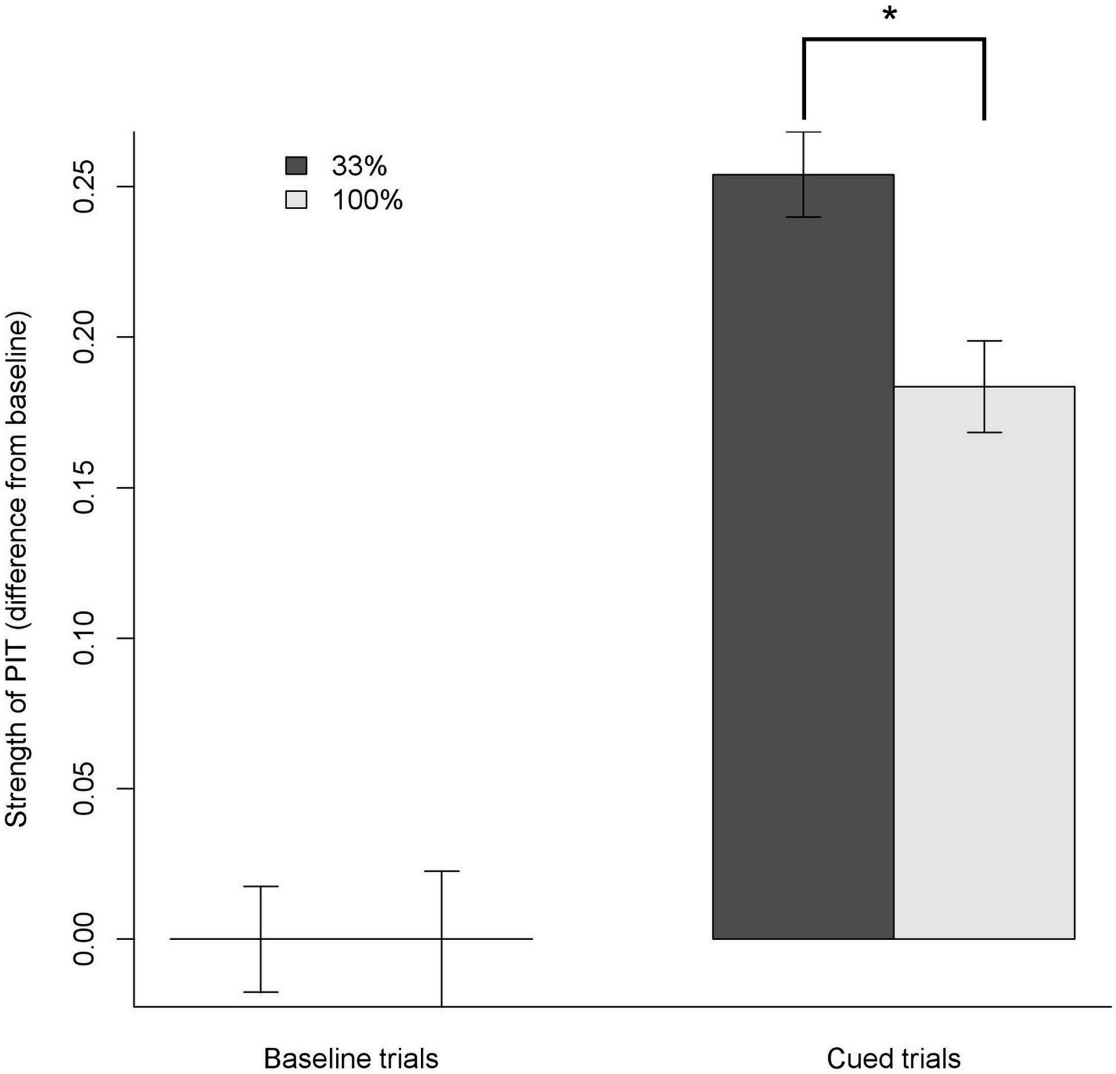
**Strength of specific PIT effect: proportion of choice obtained by subtracting the average baseline from all trials**. Positive values mean shifting the responses toward the action sharing the same outcome as the cue. During cued trials, both 33% and 100% group showed a clear specific PIT effect, shifting their choices to the same outcome as the cue by about 25% and 18%, respectively. In the 100% group, the specific PIT effect was smaller (*p < 0.001).

For each participant, we first calculated the average proportion of left presses during the 15 baseline trials (Equations 1, 2) and then subtracted this average baseline (Equation 2) from the proportion of presses during CUED trials (3). Differences obtained during the CUED trials with the visual cue associated to the same outcome of the right response were considered as having the opposite sign, so that all CUED data were positive when the cue increased the proportion of presses toward its corresponding button (see Figure 3).

We analyzed the PIT index using an ANOVA with Probability and Cued condition as factors. Both Cued, Probability and their interaction were significant (all *p* < 0.05). *Post-hoc* tests revealed that both 33% and 100% Cued trials had a PIT index significantly greater than the baseline; however, in 100% Cued trials the PIT index was smaller than in the 33% Cued trials (all *p* < 0.001). The estimated size of the Cued factor was 25.4% (95% confidence interval [20.9%; 29.9%]), meaning that in the Cued trials participants shifted their baseline proportion of responding by about 25 points toward the congruent action (e.g., from a baseline of 50% of presses on the left button to 75% of presses on the left button when the cue associated to the food congruent to the left button was displayed). The size of the interaction between the Cued factor and the Probability was –7.1% (95% confidence interval: [–14.0; –0.1%]), meaning that on average the effect of Cued trials in shifting the proportion of presses was around 7% points weaker in the 100% probability group.

## 4. Discussion

In the past, other studies have tested how PIT varies as a function of the instrumental schedule (Meltzer and Hamm, 1974b, 1978; Edgar et al., 1981; Lovibond, 1983; Holland, 2004; Wiltgen et al., 2012). These studies have found that schedules with lower baseline rates support stronger PIT (Meltzer and Hamm, 1974b, 1978; Edgar et al., 1981), which is also compatible with the observation that PIT is more easily observed after a period of extinction (Dickinson et al., 2000). Also in line with these results, Lovibond (1983) found that CS presentation tended to increase lever pressing in ratio schedules only during pauses of responding. Holland (2004) found that longer training leads to the expression of stronger PIT and Wiltgen et al. (2012) showed that training with an interval schedule expressed more PIT compared to a ratio-schedule with a similar baseline rate. However, none of these studies directly investigated the relationship between the instrumental reward probability and the strength of specific PIT. We may hypothesize that leaner schedules, which lead to lower baseline rates and more PIT (Meltzer and Hamm, 1974b, 1978; Edgar et al., 1981) do so because of a lower contingency, in line with our hypothesis that specific PIT should be stronger when reward probability is low. However, these studies used a single lever PIT paradigm with only one instrumental reinforcer, so they did not measure the CS enhancement of responding to the same outcome as the CS (specific PIT). Even if the CS and the instrumental reinforcer are the same, studies using a single lever PIT paradigm do not measure specific PIT, but a general PIT effect (Corbit and Balleine, 2005). The general PIT effect has a separate neural substrate compared to the specific PIT effect and might be also functionally different (Cartoni et al., 2013). Wiltgen et al. (2012) is the only study which used two different outcomes, thus allowing for the detection of specific PIT; however behavioral results showed that the CS increased instrumental responding in a non-outcome specific manner (general PIT). Besides, we did not find differences in baseline responding between the 100% and 33% group, so our current results cannot be explained by the above findings.

The experimental results presented in our study show a relationship between instrumental reward probability and the strength of specific PIT effect. To our knowledge, this is the first demonstration of such relationship with an explicit manipulation of the instrumental training reward probability. As we expected, specific PIT was weaker when the reward probability was higher. In other words, when participants had a higher probability of winning a reward, they were less affected by the presence of cues associated with it. Vice versa, in the condition in which participants had a lower probability of winning a reward, the presence of cues had more influence on their choice.

This relationship is in line with proposals that specific PIT works by signaling the efficacy of the instrumental actions (i.e., their probability of reward) (Cartoni et al., 2013; Hogarth et al., 2013, 2014). This can be contrasted with other proposals such as a simple ideomotor S-O-R chain (e.g., see de Wit and Dickinson, 2009, for specific PIT as S-O-R in goal-directed behavior) or the mediated S-R account (Cohen-Hatton et al., 2013). In the ideomotor account, the Pavlovian stimulus (S) simply evokes its associated outcome (O) that in turns elicits the corresponding action (R) through an O-R association learned during instrumental training. This S-O-R hypothesis can account for different instrumental contingencies by positing that different contingencies change the strength of the O-R relationship. In our case, one might then expect that in the 100% condition the O-R association should be stronger (Elsner and Hommel, 2004), thus eliciting more specific PIT, not less. The S-O-R hypothesis does not account for the integration of CS predictive information into the instrumental contingencies; it simply treats the CS as evoking the O memory and then the normal ideomotor O-R process follows. In the mediated S-R account, during the instrumental training the sight of the reward (O) evokes the memory of the Pavlovian stimulus (S). This memory is then associated with the instrumental response (R) creating an S-R association even if the Pavlovian stimulus is not actually present during the instrumental training. Again, in the mediated S-R account one would expect that a 100% contingency giving more rewards (O) would provide more occasions to evoke the Pavlovian memory and thus form a stronger S-R. So the mediated S-R account would also predict a stronger PIT in the 100% contingency group, which is the opposite of what we found.

In associative terms, our results would be more in line with a hierarchical S-(R-O) association where the presence of S modulates the expected efficacy (reward probability) of a R-O contingency. In this view the cues (S) work as “occasion setters” that signal when the instrumental contingency (R-O) is in effect (i.e., is likely to produce the outcome). Thus, in specific PIT the Pavlovian cues would work as instrumental discriminative stimuli, even if they are not explicitly trained as such, since they are not present in the instrumental training sessions. Indeed discriminative stimuli do develop such S-(R-O) relationships (Bradfield and Balleine, 2013). Recent experimental data from Hogarth et al. (2014) provides support to these hierarchical relationships in specific PIT. Specific PIT is known to be resistant to the extinction of the binary Pavlovian S-O associations (Delamater, 1996), unless these associations have had a short training (Delamater, 2012). In Hogarth et al. (2014) it was shown that specific PIT can be more readily abolished by targeting the hierarchical S-(R-O) relationship rather than the S-O association. This can be done by using either discriminative extinction training or by explicit verbal instructions to the participants stating that the cues would not provide information about the most likely rewarded action (Hogarth et al., 2014).

Despite the fact that our results are in line with the idea that specific PIT works by enhancing the participants estimates of the reward probability, we still observed a clear specific PIT effect even in the 100% probability group, where reward probability is already at maximum. A possible explanation of this is that even if they experienced a 100% reward probability during the instrumental phase, the participants still considered the reward as not fully certain in the test phase, thus allowing some room for the cue to have an effect. In particular, this uncertainty could be the result of the test phase being carried out in extinction. Even if it was only a nominal extinction and participants were explicitly told the rewards were still being earned as in the instrumental phase, the removal of the visual feedback of the reward might still have caused some uncertainty about the likelihood of reward delivery, at a conscious or unconscious level. Indeed, in animal studies it was found that PIT is more easily detected after a period of extinction (Dickinson et al., 2000).

In alternative, it might simply be that there are additional factors beyond reward probability involved in the specific PIT effect. It will be interesting to investigate in future studies how much the reduction of uncertainty of reward contributes to the specific PIT effect compared to other possible factors not involving the predictive validity of cues, such as facilitation effects by cued retrieval of the outcome (e.g., a S-O-R account).

A limitation of this study is that the 100% probability group received more rewards than the 33% group, so the number of rewards is a possible confound. Future studies might include a group with low probability but longer training to equate the number of rewards received. The relationship between outcome probability and specific PIT effect found in this study should be further investigated as here we tested only two possible probabilities. Using a wider set of probabilities should give a better account of how the size of the specific PIT effect varies by changing the instrumental contingency and also give a better picture of the relevance of this factor. Despite these limitations, this study represents a first step into the exploration of the relationship between the instrumental contingency strength and the specific PIT effect.

## Conflict of interest statement

The authors declare that the research was conducted in the absence of any commercial or financial relationships that could be construed as a potential conflict of interest.
